# Functional Near-Infrared Spectroscopy-Based Computer-Aided Diagnosis of Major Depressive Disorder Using Convolutional Neural Network with a New Channel Embedding Layer Considering Inter-Hemispheric Asymmetry in Prefrontal Hemodynamic Responses

**DOI:** 10.1155/2024/4459867

**Published:** 2024-07-14

**Authors:** Kyeonggu Lee, Jinuk Kwon, Minyoung Chun, JongKwan Choi, Seung-Hwan Lee, Chang-Hwan Im

**Affiliations:** ^1^Department of Electronic Engineering, Hanyang University, Seoul, Republic of Korea; ^2^OBELAB Inc., Seoul, Republic of Korea; ^3^Department of Psychiatry, Clinical Emotion and Cognition Research Laboratory, Inje University, Goyang, Republic of Korea; ^4^Ilsan Paik Hospital, Inje University College of Medicine, Juhwa-ro 170, Ilsanseo-Gu, Goyang 10370, Republic of Korea; ^5^Department of Biomedical Engineering, Hanyang University, Seoul, Republic of Korea

## Abstract

**Background:**

Functional near-infrared spectroscopy (fNIRS) is being extensively explored as a potential primary screening tool for major depressive disorder (MDD) because of its portability, cost-effectiveness, and low susceptibility to motion artifacts. However, the fNIRS-based computer-aided diagnosis (CAD) of MDD using deep learning methods has rarely been studied. In this study, we propose a novel deep learning framework based on a convolutional neural network (CNN) for the fNIRS-based CAD of MDD with high accuracy.

**Materials and Methods:**

The fNIRS data of participants—48 patients with MDD and 68 healthy controls (HCs)—were obtained while they performed a Stroop task. The hemodynamic responses calculated from the preprocessed fNIRS data were used as inputs to the proposed CNN model with an ensemble CNN architecture, comprising three 1D depth-wise convolutional layers specifically designed to reflect interhemispheric asymmetry in hemodynamic responses between patients with MDD and HCs, which is known to be a distinct characteristic in previous MDD studies. The performance of the proposed model was evaluated using a leave-one-subject-out cross-validation strategy and compared with those of conventional machine learning and CNN models.

**Results:**

The proposed model exhibited a high accuracy, sensitivity, and specificity of 84.48%, 83.33%, and 85.29%, respectively. The accuracies of conventional machine learning algorithms—shrinkage linear discriminator analysis, regularized support vector machine, EEGNet, and ShallowConvNet—were 73.28%, 74.14%, 62.93%, and 62.07%, respectively.

**Conclusions:**

In conclusion, the proposed deep learning model can differentiate between the patients with MDD and HCs more accurately than the conventional models, demonstrating its applicability in fNIRS-based CAD systems.

## 1. Introduction

Major depressive disorder (MDD) is one of the leading causes of global disease burden [1], with reports indicating that ~20% of the world's population experiences it at least once in their lifetime [2]. The COVID-19 pandemic and severe climate changes have gradually increased the prevalence of MDD [3, 4] and its emergence as a critical social issue worldwide. Accurately diagnosing MDD is necessary to mitigate its social impact, particularly for timely treatment planning [5, 6]. However, the current conventional MDD diagnosis methods based on the Diagnostic and Statistical Manual of Mental Disorders (DSM-IV or DSM-5) [7, 8] sometimes lead to misdiagnosis because these face-to-face interviews rely solely on the subjective responses of patients for diagnosis [9, 10].

To enhance the objectivity and reliability of MDD diagnosis, neuroimaging techniques, such as electroencephalography (EEG), functional magnetic resonance imaging, computerized tomography, functional near-infrared spectroscopy (fNIRS), and positron emission tomography, have been widely employed [11, 12, 13, 14, 15]. With rapid advancements in wearable technologies, EEG and fNIRS have emerged as new tools for the screening and early self-diagnosis of psychiatric diseases, including MDD [16, 17]. Among the various neuroimaging modalities, portable fNIRS systems, which generally measure prefrontal brain activity, have shown several advantages, such as portability, reduced sensitivity to physiological artifacts, and enhanced user-friendliness. Various fNIRS studies have been conducted to differentiate hemodynamic responses between individuals with MDD and healthy control (HC) groups [18, 19, 20, 21, 22] for the implementation of fNIRS-based MDD screening systems in daily life [23].

Previous studies reported that on analyzing the differences in MDD and HC groups while performing a cognitive task, two notable characteristics of fNIRS signals were observed in MDD patients: lower activation in the prefrontal area [18, 19, 20] and increased hemispheric asymmetry of hemodynamic responses [21, 22]. These findings have led to the application of machine learning methods, such as support vector machine (SVM), k-nearest neighbors, and decision trees, for the computer-aided diagnosis (CAD) of MDD. The conventional machine learning approaches employ various handcrafted features that reflect the hemodynamic disparities between the MDD and HC groups, resulting in high classification accuracy, sometimes exceeding 80% [24, 25, 26, 27]. However, employing handcrafted features for CAD of psychiatric diseases generally requires careful engineering and domain expertise, which is a time-consuming procedure [28]. In addition, the selection of appropriate features ensuring generalized performance is challenging [29]. Alternatively, deep learning approaches that can address the issues related to handcrafted features are promising because they can automatically extract significant features from raw or minimally preprocessed input data [29].

A few fNIRS studies have applied deep learning models to classify HCs and patients with MDD. Wang et al. [30] demonstrated a high classification accuracy of 90% by utilizing a correlation map as an input for AlexNet. Extending this research with the same dataset, Yu et al. [27] achieved an accuracy of 87.5% by employing a coherence matrix as the input for a graph neural network. However, these two studies categorized the groups based on a patient health questionnaire-9 (PHQ-9) score and not on a clinical diagnosis by a psychiatrist. Therefore, it is difficult to stipulate that these studies target patients with MDD. Additionally, Wang et al. [31] achieved an 89.1% accuracy using a spectrum map of wavelet transform as an input for a parallel convolutional neural network (CNN) model; however, this study used a small dataset comprising only 49 participants, which included 21 MDD patients. There exists only one study [32] that utilized a large dataset comprising more than 100 participants and reported a classification accuracy of 80.9% using the multiple channels network. Previous deep learning studies have mostly utilized handcrafted features or architectures that were not specifically designed for CAD of MDD. Therefore, it is necessary to further explore effective deep learning frameworks that are specifically tailored for higher-accuracy fNIRS-based CAD systems targeting MDD.

In this study, we propose a new deep learning framework specifically designed for fNIRS-based CAD of MDD. The first convolutional layer in our proposed deep learning framework extracts temporal features, thereby capturing the differences in the temporal patterns of fNIRS data between patients with MDD and HCs during the performance of a cognitive task. In addition, we specially designed a channel-embedding convolutional layer to consider the hemispheric asymmetry of brain activation, which is known to be a significant factor in diagnosing MDD in conventional handcrafted feature-based machine learning studies. To the best of our knowledge, this is the first attempt at designing a deep learning architecture that reflects the asymmetric characteristics of hemodynamic responses between patients with MDD and HCs. The proposed deep learning architecture was validated using fNIRS data acquired from 116 participants (48 patients with MDD and 68 HCs) during a Stroop task. The effectiveness of the proposed model was evaluated by comparing its performance with that of machine learning models with handcrafted features and two conventional deep learning models.

## 2. Methods and Materials

### 2.1. Participants

fNIRS data were acquired from 116 participants (48 patients with MDD and 68 HCs) at the Inje University Ilsan Paik Hospital (Goyang, South Korea). Participants with sensory impairment, epilepsy, brain injury, or a history of psychotic symptoms were excluded from the study. MDD was diagnosed based on DSM-5 by a board-certified psychiatrist. The depression severity of all participants was assessed using a self-reported inventory such as the Korean version of the Beck's Depression Inventory-II (BDI-II) [33], the state anxiety section (SAI) [34], and Hamilton's depression rating scale (HAM-D) [35, 36].

Almost all patients with MDD enrolled in this study were taking antidepressants. While controlling for medication would have yielded purer results, significant ethical and practical challenges exist because removing or altering prescribed medications could exacerbate symptoms or harm the patients, making such controls ethically unacceptable and practically difficult to implement. Previous studies have reported that the effect of antidepressants on fNIRS data is minor [19] or that none of the medications and clinical variables showed any association with fNIRS-derived indicators [37]. Conversely, one study showed that the defined daily dose of antidepressants was statistically related to fNIRS data on a specific channel; however, this channel was located in the right temporoparietal region, which is not covered by the fNIRS device used in this study. Based on these, we speculate that antidepressants have limited impacts on the classification results of the machine learning model.

Demographic data, including sex, age, and educational level (years of completed formal education), were compared between patients with MDD and HCs. Detailed demographic information and BDI-II, SAI, and HAM-D scores of patients with MDD and HCs are summarized in Table 1. All procedures were approved by the Institutional Review Board of the Inje University Ilsan Paik Hospital (2017-10-013). Informed consent was obtained from all the participants prior to the experiments.

### 2.2. fNIRS Recording and Preprocessing

The fNIRS data were acquired while the participants engaged in a Stroop task, which included three 30-s task blocks, interspersed with 30-s resting intervals. During the task block, participants were presented with one of the seven words with different font colors (red, blue, yellow, gray, orange, pink, or green) randomly and consecutively on a monitor. The font color of each word did not match the meaning of the corresponding word. The participants were instructed to identify the font color rather than the color indicated by the word. The graphical illustration for the fNIRS measurement paradigm is presented in Figure S1.

A high-density NIRS recording device, NIRSIT (OBELAB; Seoul, Korea), was used to measure the fNIRS signals. The device was equipped with 24 dual-wavelength laser diodes (780/850 nm) and 32 photodetectors positioned 3 cm apart from each other [38]. The fNIRS signals from the 68 channels were acquired at a sampling rate of 8.138 Hz.

Raw fNIRS data were preprocessed using MATLAB 2020b (MathWorks; Natick, MA, USA) with functions implemented in the BBCI toolbox (https://github.com/bbci/bbci_public). The optical density signals were low-pass filtered at 0.5 Hz using a 6th-order Butterworth zero-phase filter to remove experimental noise and instrumental noise [39]. The concentration changes in oxygenated (*Δ*HbO) and deoxygenated (*Δ*HbR) hemoglobins for each channel were extracted from the optical density signals using the modified Beer–Lambert law (MBLL) [40]. The extracted *Δ*HbO and *Δ*HbR were bandpass filtered at 0.01–0.3 Hz using a 6th-order Butterworth zero-phase filter to remove physiological noises [41]. The *Δ*HbO and *Δ*HbR were then divided into 30-s segments, corresponding to the execution time of the task blocks, resulting in three segments per participant. Baseline correction for each segment was conducted by subtracting the temporal mean value within the interval of −1 to 0 s from the segment.

### 2.3. Proposed Deep Learning Architecture

Using preprocessed *Δ*HbO and *Δ*HbR, a vector-based analysis was conducted to determine changes in cerebral blood volume (*Δ*CBV) and cerebral oxygen exchange (*Δ*COE). Additionally, phase indicators, including the magnitude (|*L*|) and angle (*k*) of the vectors, were calculated (Table 2). These indicators serve as quantitative measures of oxygen metabolism [42]. Subsequently, the six hemodynamic response indicators (*Δ*HbO, *Δ*HbR, *Δ*CBV, *Δ*COE, |*L*|, and *k*) were averaged across the three segments and used as inputs for the deep learning models. The examples of these six indicators are illustrated in Figure S2.

**Figure 1:**
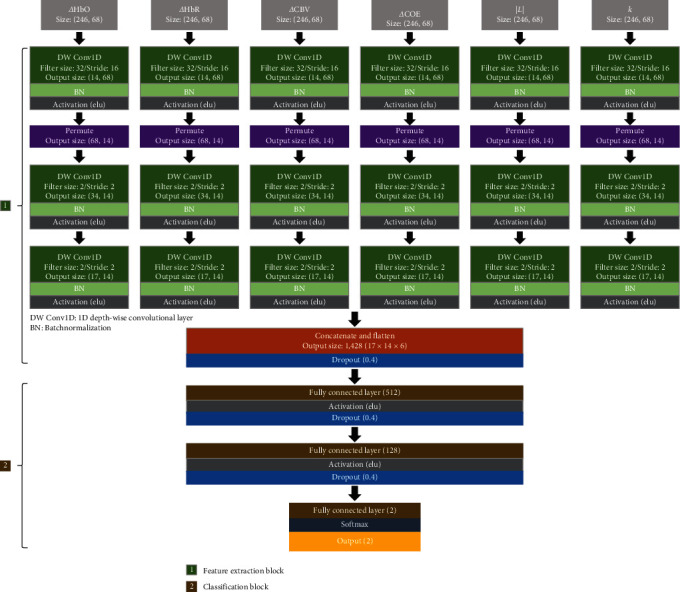
Overall architecture of the proposed deep learning model.

As shown in Figure 1, the proposed deep learning model extracts features from six hemodynamic response indicators by employing an ensemble approach and three 1D depthwise convolutional layers (DW Conv1D). The initial layer (temporal layer) extracts temporal features independently for each channel, and the second and third layers (spatial layers) extract spatial features by leveraging the spatial configuration of the channels, thereby facilitating the automatic formation of spatiotemporal representations.

**Figure 2:**
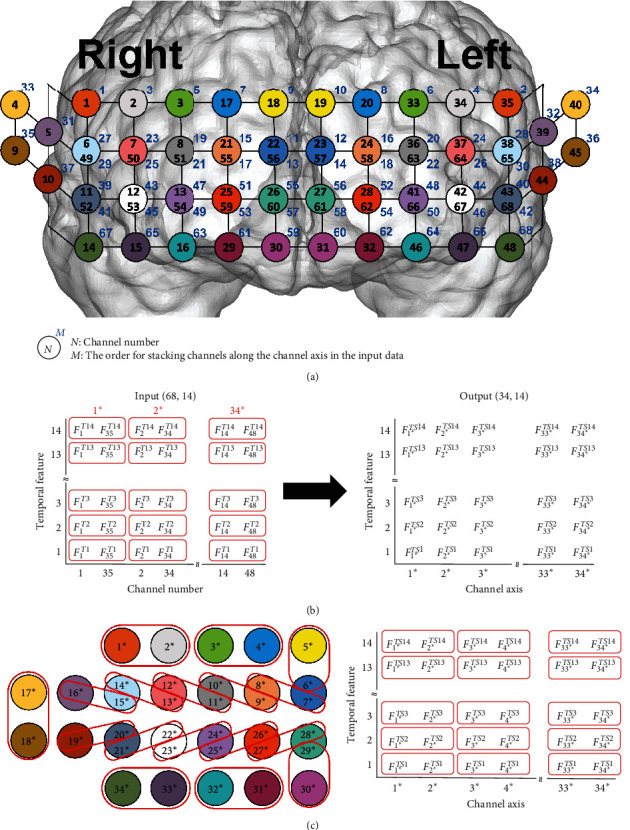
Description of the procedure for the extraction of spatial features: (a) the channel configuration and the order for stacking channels along the channel axis in the input data; (b) the procedure for the extraction of spatial features from pairs of symmetric channels; (c) the procedure for the extraction of spatial features from two adjacent channels. The notation “*⁣*^*∗*^” after each number refers to the assigned channel numbers after passing through the second spatial layer.

To extract spatial features, the sequence of channels in the input data was first reorganized, as depicted in Figure 2(a). The temporal layer utilized a filter size of 32 and filter stride of 16, enabling feature calculation over a 4-s window with a 2-s overlap. Each spatial layer used a filter size of 2 and stride of 2, with the first layer extracting features from pairs of symmetrical channels (Figure 2(b)) and the second layer capturing features from two adjacent channels (Figure 2(c)). This channel-embedding strategy was specifically designed for the deep learning model to account for hemispheric asymmetry in hemodynamic responses between patients with MDD and HCs. This approach also aims to effectively reduce feature dimensionality while preserving the integrity of channel configuration information. Following feature extraction, the features derived from the six hemodynamic response indicators were concatenated and flattened. Finally, the classification block, which comprises three dense layers, produces classification outcomes.

To highlight the significance of the systematic spatiotemporal feature extraction proposed in this study, the performance of the proposed architecture was evaluated in terms of accuracy, sensitivity, and specificity and compared with three alternative feature extraction strategies: a deep learning framework that extracts only temporal features without considering spatial features (C1), a deep learning model that prioritizes the extraction of spatial features before the extraction of temporal features (C2), and a deep learning architecture identical to the proposed architecture but with the channel order randomized in the input data (C3). Notably, the network architectures for C1 and C2 were identical to the proposed deep learning model, with the only difference being in the feature extraction block. The specific network architectures of the feature extraction blocks for C1 and C2 are presented in Table 3. The randomized channel order used for the input data in C3 is illustrated in Figure S3 in the supplementary material.

### 2.4. Conventional Machine Learning and Deep Learning Algorithms

Conventional machine learning and deep learning algorithms were applied to the acquired fNIRS data for comparisons with the proposed deep learning model. The evaluated machine learning algorithms, which utilized handcrafted features, were shrinkage linear discriminant analysis (sLDA) and regularized SVM (rSVM). The tested deep learning algorithms included EEGNet and ShallowConvNet.

#### 2.4.1. sLDA and rSVM

sLDA and rSVM were implemented using functions from the statistic and machine learning Toolbox and the BBCI toolbox of MATLAB 2020b, respectively. These methods are particularly advantageous when the dataset has a large number of features compared with the number of training samples [43, 44]. rSVM was implemented using the fitclinear() function, which was designed to train binary classification models with high-dimensional features using ridge regularization. sLDA is beneficial for enhancing the estimation of the covariance matrix in situations where the sample-to-feature ratio is low. To train these machine learning models, six statistical metrics (mean, variance, kurtosis, skewness, peak value, and time to peak) were extracted from each of the six hemodynamic indicators (*Δ*HbO, *Δ*HbR, *Δ*CBV, *Δ*COE, |*L*|, and *k*), resulting in 2,448 candidate features (6 indicators × 68 channels × 6 features).

#### 2.4.2. ShallowConvNet and EEGNet

ShallowConvNet and EEGNet are widely used CNN models renowned for their efficacy in extracting spatiotemporal features from EEG data [45]. These EEG-based deep learning models were selected for comparison with the proposed model because of their architectural similarities, including both temporal and spatial convolutional layers, similar to those in the proposed deep learning framework. Furthermore, a prior fNIRS-based brain–computer interface study [46] highlighted the superior performance of EEGNet over sLDA, reinforcing the rationale for their inclusion in conventional deep learning models for comparison.

To apply the ensemble approach to ShallowConvNet and EEGNet, the original classifier stage, which consisted of a dense layer with two units, was omitted. The following classification block, which merges features from the six hemodynamic response indicators via a concatenated layer, is designed to be identical to that of the proposed model.  Table 4 presents a comprehensive description of the feature extraction blocks for both EEGNet and ShallowConvNet. The hyperparameters of EEGNet and ShallowConvNet were adjusted to ensure that the number of features fed into the classification block matched with that of the proposed model.

### 2.5. Performance Evaluation

The performances of the machine learning and deep learning algorithms were evaluated using the leave-one-subject-out cross-validation (LOSO-CV) approach. In this setup, each round of validation involved 115 training samples and one test sample, and this process was repeated across all 116 iterations to evaluate the performance in terms of accuracy, sensitivity, and specificity. For machine learning algorithms (sLDA and rSVM) that rely on handcrafted features, feature normalization and selection were implemented. Feature normalization was performed using the *z*-score normalization for the training data, and the test data were normalized to the mean and variance derived from the training set. Feature selection was performed at each LOSO-CV iteration using the Fisher score—a prominent filter method in supervised feature selection—to identify and select the optimal subset of features [47]. The “*N*-feature accuracy” was evaluated by averaging the outcomes across all LOSO-CV iterations for sets of *N* features, with *N* varying from 1 to 50 [48]. The highest value obtained from the “*N*-feature accuracy” assessments was designated as the classification accuracy for the machine learning algorithms.

For the deep learning algorithms, including the proposed model, C1, C2, C3, ShallowConvNet, and EEGNet, the number of training epochs was determined by identifying the point at which both training accuracy and loss, averaged over 116 LOSO-CV iterations, reached convergence. Specifically, as illustrated in Figure S4 in the supplementary material, this was set to 10 epochs for C1; 20 epochs for the proposed model, C2, C3, and EEGNet, respectively; and 50 epochs for ShallowConvNet. Training and evaluation were conducted on a desktop computer equipped with an Intel Core i7-7700 processor, 32 GB RAM, and an NVIDIA GeForce RTX 2060 Ti GPU, utilizing Keras (https://keras.io) with a TensorFlow backend. Each deep learning model was trained to minimize the categorical cross-entropy loss function with a batch size of 32 and the Adamax optimizer [49], at a default learning rate of 0.001 to ensure fair comparison under uniform conditions. A fixed random seed of 0 was used to maintain consistency across the training sessions. To address potential classification biases arising from class imbalances, the *class_weight* parameter was empirically adjusted to {0 : 0.9, 1 : 1.0} [50, 51], where “0” denotes the HC class and “1” represents the MDD class.

## 3. Results

The performances of the proposed deep learning model and its variants, C1, C2, and C3, for CAD of MDD are presented in Table 5. The proposed model achieved an accuracy of 84.48%, with sensitivity and specificity rates of 83.33% and 85.29%, respectively. This model outperformed C1, C2, and C3 in terms of accuracy and demonstrated a more balanced performance for sensitivity and specificity. Specifically, models C1 and C3, which overlooked the spatial characteristics of fNIRS data associated with MDD, exhibited higher specificity than sensitivity. Conversely, model C2, which did not extract temporal information independently for each channel, showed higher sensitivity than specificity.

Table 6 presents the performance metrics of conventional machine learning and deep learning algorithms. The proposed deep learning model outperformed those of the conventional algorithms in accuracy by ~14%. Among the conventional algorithms, sLDA and rSVM demonstrated superior performance to EEGNet and ShallowConvNet, in terms of both accuracy and the balance between sensitivity and specificity. The performances of EEGNet and ShallowConvNet were comparable, with both models exhibiting a notable preference for specificity over sensitivity, with a bias exceeding 30%.

**Figure 3:**
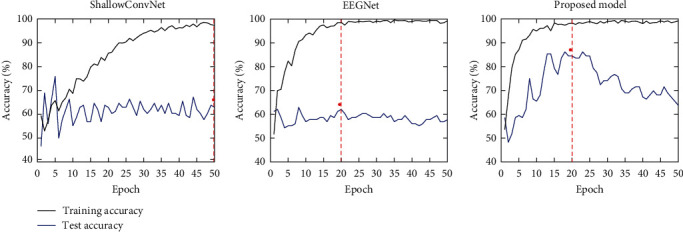
Training accuracy and test accuracy as a function of epoch numbers for ShallowConvNet, EEGNet, and proposed model. The red dashed lines denote the number of epochs used to evaluate the accuracy of each deep learning model.

Figure 3 illustrates a comparison of the test accuracy with respect to the number of epochs to assess overfitting in three deep learning models: the proposed model, EEGNet, and ShallowConvNet. For EEGNet and ShallowConvNet, although the training accuracy approached ~100% with increasing epochs, test accuracy remained below 70%, indicating early onset of overfitting in these models. Conversely, the test accuracy of the proposed model increased to 86.21% by the 18th epoch, aligned closely with the point where training accuracy converged, and then decreased as epochs increased owing to overfitting after 25 epochs. Overall, the proposed model demonstrates a higher average test accuracy and lower susceptibility to overfitting than EEGNet and ShallowConvNet.

## 4. Discussion

In this study, we proposed a deep learning model that is tailored exclusively for the fNIRS-based CAD of patients with MDD. The cornerstone of our model is its ability to account for hemispheric asymmetry commonly observed in patients with MDD, which is achieved through a unique channel-embedding strategy that pairs symmetric channels. The initial DW Conv1D layer of the proposed model was designed to extract temporal features independently from each channel, whereas the subsequent DW Conv1D layer was adept at capturing spatial features that reflected hemispheric asymmetry in the hemodynamic responses. Our results demonstrate that the proposed model surpasses not only its three altered versions (C1, C2, and C3) but also the conventional machine learning and deep learning algorithms in terms of accuracy and in maintaining a balance between sensitivity and specificity.

We compared the proposed framework with three alternative models to understand the reasons for our model achieving the highest accuracy while maintaining a balance between sensitivity and specificity. The C1 architecture lacks a convolutional layer for utilizing spatial information, whereas the C3 paradigm extracts spatial features without considering the actual channel configuration. In both cases, the specificity exceeded the sensitivity. This finding is in line with a recent study that employed a similar model architecture to C1 [32], which also reported higher specificity than sensitivity (accuracy, 80.9%; sensitivity, 75.9%; and specificity, 85.2%). Conversely, the C2 paradigm, which did not consider the temporal information of each channel, resulted in higher sensitivity than specificity. The outcomes from C1 and C2 suggest that our proposed model achieves an unbiased nature by counterbalancing the potential bias of the decision-making boundary toward HCs in the temporal layer by introducing a systematic approach to extracting spatial information. Furthermore, a comparison between our model and C3 underscores the importance of systematic spatial information extraction in enhancing the overall efficacy of CAD for MDD.

The proposed model demonstrated superior accuracy over conventional machine learning algorithms, achieving ~14% higher accuracy without relying on handcrafted feature extraction. This marks a significant milestone in fNIRS-based CAD systems for MDD, showcasing, to the best of our knowledge, that features automatically extracted from minimally preprocessed hemodynamic responses using a deep learning model can outperform those derived from handcrafted features in machine learning algorithms. Furthermore, the proposed model surpassed existing deep learning algorithms in terms of performance and exhibited a lower tendency toward overfitting. This advantage is partly because the spatial layer in EEGNet and ShallowConvNet compresses temporal features from all channels into a single value over a specific time range, potentially leading to a loss of spatial information, as suggested by Schirrmeister et al. [52] and Lawhern et al. [53]. This loss of spatial information in EEGNet and ShallowConvNet, similarly observed in the C1 and C3 paradigms, could explain their significantly higher specificity than sensitivity.

The machine learning and deep learning models tested in this study showed relatively lower performance compared to those in previous studies. We hypothesized that the Stroop task might be less suited to discriminating patients with MDD from HCs than the verbal fluency task (VFT) because most previous fNIRS-based studies for the diagnosis of MDD have employed the VFT paradigm [20]. While VFT has been widely used for the machine learning-based diagnosis of MDD [54], no study has employed the Stroop task for the same purpose. Nevertheless, it is noteworthy that the proposed deep learning model showed notable performance even with the Stroop task, suggesting that the Stroop task can also provide valuable data if analyzed with advanced deep learning techniques.

In this study, conventional deep learning models exhibited lower accuracies than machine learning models. We assumed that the small amount of training data used in this study resulted in higher accuracy for the machine learning models than for the deep learning models. Previous studies support this observation, indicating that machine learning algorithms often outperform deep learning models when dealing with small datasets [55, 56]. Although our proposed model surpassed the machine learning models in terms of accuracy, even with a small dataset, acquiring more data can further enhance the classification performance of the proposed model. In addition, machine learning algorithms can be used to interpret the results using the selected handcrafted features; however, there is no theoretical explanation for the results of the proposed model, which is a so-called black-box problem [57]. Therefore, in future studies, we intend to collect more data and devise effective methods to elucidate the working mechanisms of the proposed model. It is expected that recent advancements in explainable artificial intelligence (XAI) technologies [58], such as layer-wise relevance propagation (LRP) [59] and gradient-weighted class activation mapping (grad-CAM) [60], would enable in investigating the interpretability of our deep learning model. Additionally, we plan to investigate the generalizability of the proposed deep learning model by applying it to other fNIRS data from different paradigms in which hemispheric asymmetry is generally observed [61, 62].

## 5. Conclusion

In this study, we developed a CNN-based deep learning architecture for the CAD of MDD with a new channel embedding layer to reflect interhemispheric asymmetry in prefrontal hemodynamic responses between patients with MDD and HCs, which have been known to be a distinct characteristic in previous MDD studies. The proposed model can differentiate between the patients with MDD and HCs more accurately than the conventional models, demonstrating its applicability in fNIRS-based CAD systems.

## Figures and Tables

**Table 1:** Demographics of the MDD and HC groups.

Variables	MDD (*N* = 48)	HC (*N* = 68)	*p*-Value
Age	39.21 ± 14.50	34.26 ± 12.97	0.057
Sex (male/female)	16/32	26/42	0.588
Education (years)	11.52 ± 2.48	12.93 ± 2.94	0.008
BDI-II	21.15 ± 13.81	8.59 ± 7.12	<0.001
SAI	48.65 ± 14.08	34.25 ± 8.47	<0.001
HAM_D	19.15 ± 11.85	4.90 ± 5.07	<0.001

**Table 2:** Formulas for calculating *Δ*CBV, *Δ*COE, |*L|*, and *k*.

Representation	Formula
*Δ*CBV	$\frac{\Delta \text{HbO}+\Delta \text{HbR}}{2}$
*Δ*COE	$\frac{\Delta \text{HbO}-\Delta \text{HbR}}{2}$
|*L*|	$\frac{\sqrt{\Delta {\text{HbO}}^{2}+\Delta {\text{HbR}}^{2}}}{\sqrt{2}}$
*k*	$\tan^{-1}\frac{\Delta \text{COE}}{\Delta \text{CBV}}$

**Table 3:** Network architectures of the feature extraction block of C1 and C2.

Layer	Kernel size/stride	Normalization or dropout	Output shape	Options
C1				
Input	—	—	6 × (246, 68)	—
DW Conv1D	32/16	BatchNorm	6 × (14, 68)	Padding = valid Activation = ELU
Concatenate	—	—	(14, 68, 6)	—
Flatten	—	Dropout (*p*=0.4)	5,712	—
C2				
Input	—	—	6 × (68, 246)	—
DW Conv1D	2/2	BatchNorm	6 × (34, 68)	Padding = valid Activation = ELU
DW Conv1D	2/2	BatchNorm	6 × (17, 68)	Padding = valid Activation = ELU
Permute	—	—	6 × (68, 17)	—
DW Conv1D	32/16	BatchNorm	6 × (14, 17)	Padding = valid Activation = ELU
Concatenate	—	—	(14, 17, 6)	—
Flatten	—	Dropout (*p*=0.4)	1,428	—

**Table 4:** Network architectures of the feature extraction block of ShallowConvNet and EEGNet.

Layer	Filters/kernel size/stride	Normalization or dropout	Output shape	Options
ShallowConvNet				
Input	—	—	6 × (68, 246, 1)	—
Conv2D (temporal)	1/(1, 16)/(1, 4)	—	6 × (68, 58, 1)	Padding = valid Max norm = 2
Conv2D (spatial)	17/(68, 1)/−	BatchNorm (epsilon = 1e-05, momentum = 0.1)	6 × (1, 58, 17)	Padding = valid Max norm = 2 Activation = square
AveragePooling2D	−/(1, 5)/ (1, 4)	Dropout (*p*=0.4)	6 × (1, 14, 17)	Activation = log
Concatenate	—	—	(6, 14, 17)	—
Flatten	—	—	1,428	—
EEGNet				
Input	—	—	6 × (68, 246, 1)	—
Conv2D (temporal)	1/(1, 16)/(1, 4)	BatchNorm()	6 × (68, 58, 1)	Padding = valid
DW Conv2D (spatial)	17/(1, 16)/(1, 4)	BatchNorm()	6 × (1, 58, 17)	Padding = valid Depth = 17 Max norm = 1 Activation = ELU
AveragePooling2D	−/(1, 8)/(1, 2)	Dropout (*p*=0.4)	6 × (1, 26, 17)	—
Sperable Conv2D	17/(1, 8)/(1, 1)	BatchNorm()	6 × (1, 19, 17)	Padding = valid Activation = ELU
AveragePooling2D	−/(1, 6)/(1, 1)	—	6 × (1, 14, 17)	—
Concatenate	—	—	(6, 14, 17)	—
Flatten	—	—	1,428	—

**Table 5:** Performances of the proposed model, C1, C2, and C3.

Condition	Accuracy (%)	Sensitivity (%)	Specificity (%)
C1	68.97	39.58	89.71
C2	47.41	100	10.29
C3	62.07	8.33	100
Proposed model	**84.48**	**83.33**	**85.29**

The highest results are highlighted in bold.

**Table 6:** Performances of the conventional machine learning and deep learning algorithms.

Algorithm	Accuracy (%)	Sensitivity (%)	Specificity (%)
sLDA	73.28	75	72.06
rSVM	74.14	81.25	69.12
ShallowConvNet	62.93	43.75	76.47
EEGNet	62.07	43.75	75
Proposed model	**84.48**	**83.33**	**85.29**

The highest results are highlighted in bold.

## Data Availability

The data utilized in this study are available from the corresponding author upon request.

## Supplementary Materials

Supplementary Materials Supplementary materials include methodological details supporting this study. All supplementary files are available for download and review.
