# Supplementary material for: Functional Near-Infrared Spectroscopy-Based Computer-Aided Diagnosis of Major Depressive Disorder Using Convolutional Neural Network with a New Channel Embedding Layer Considering Inter-Hemispheric Asymmetry in Prefrontal Hemodynamic Responses
Source: Depress Anxiety. 2024 Jul 14;2024:4459867. doi: 10.1155/2024/4459867 (PMC11918759; doi:10.1155/2024/4459867)
Supplement: Supplementary Materials — include methodological details supporting this study. All supplementary files are available for download and review. [file 4459867.f1.pdf]

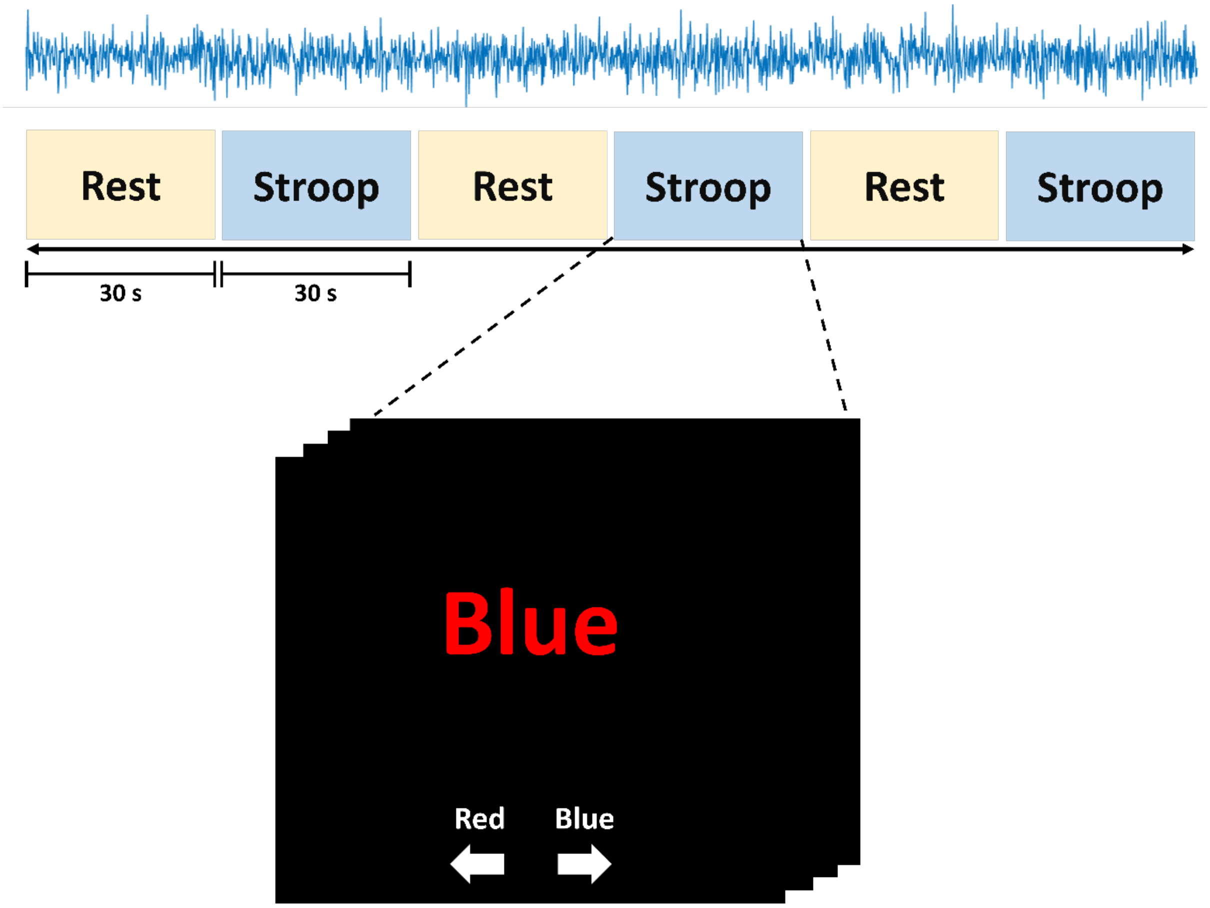

Figure S1. Experimental paradigm. fNIRS data were acquired during the three 30-s Stroop task blocks, each preceded by a 30-second resting interval. In the Stroop task block, for example, the word "blue" might be written in red ink. On the bottom row, two color words ("blue" and "red") appeared in white ink on the left and right sides. Participants were tasked with determining which of the two bottom colors matched the ink color of the top word, making their selection by pressing the left or right arrow keys on a keyboard.

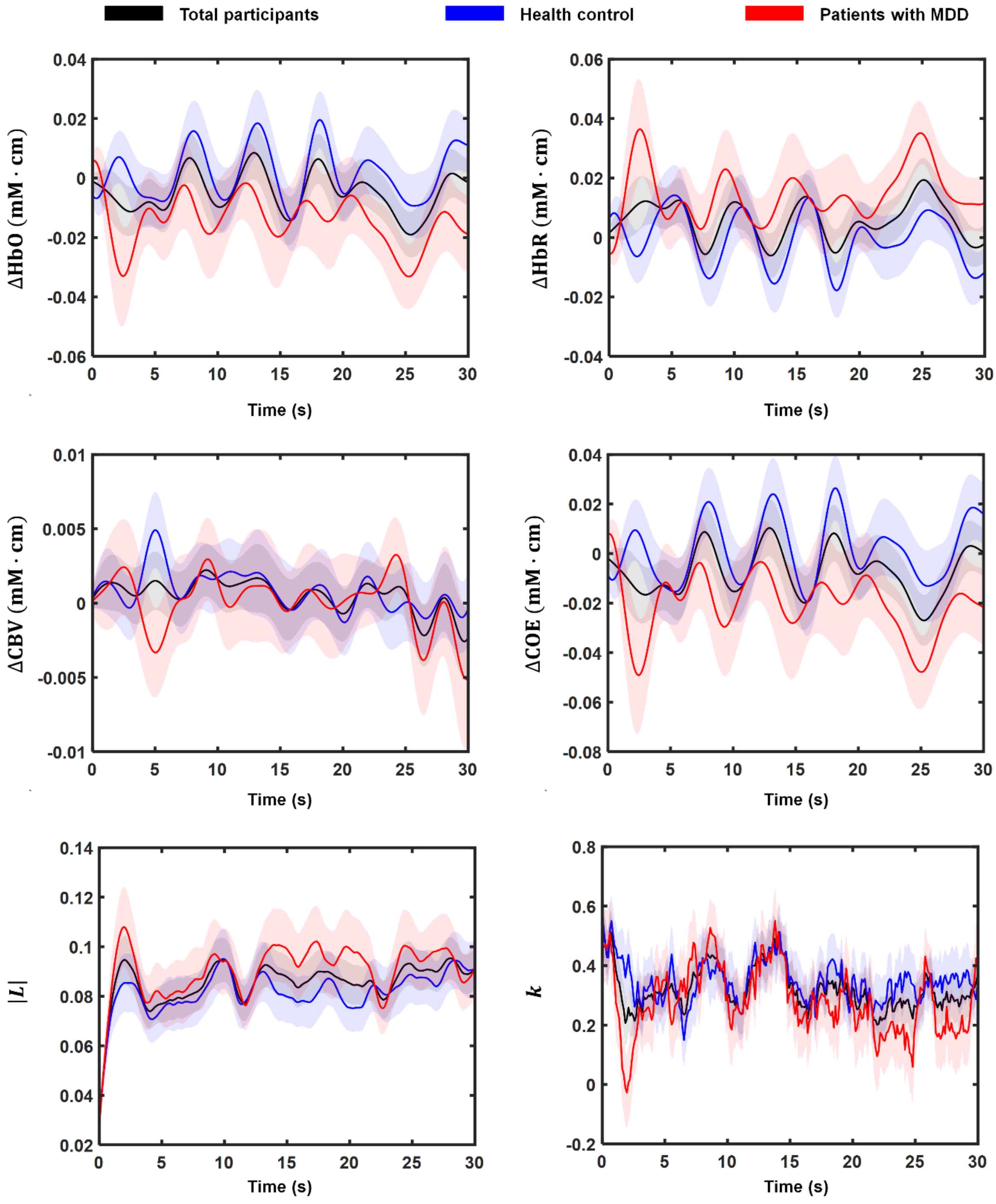

Figure S2. Six hemodynamic response indicators derived from channel 4. These indicators were averaged across all participants, healthy controls, and patients with MDD. The shade areas denote the standard error.

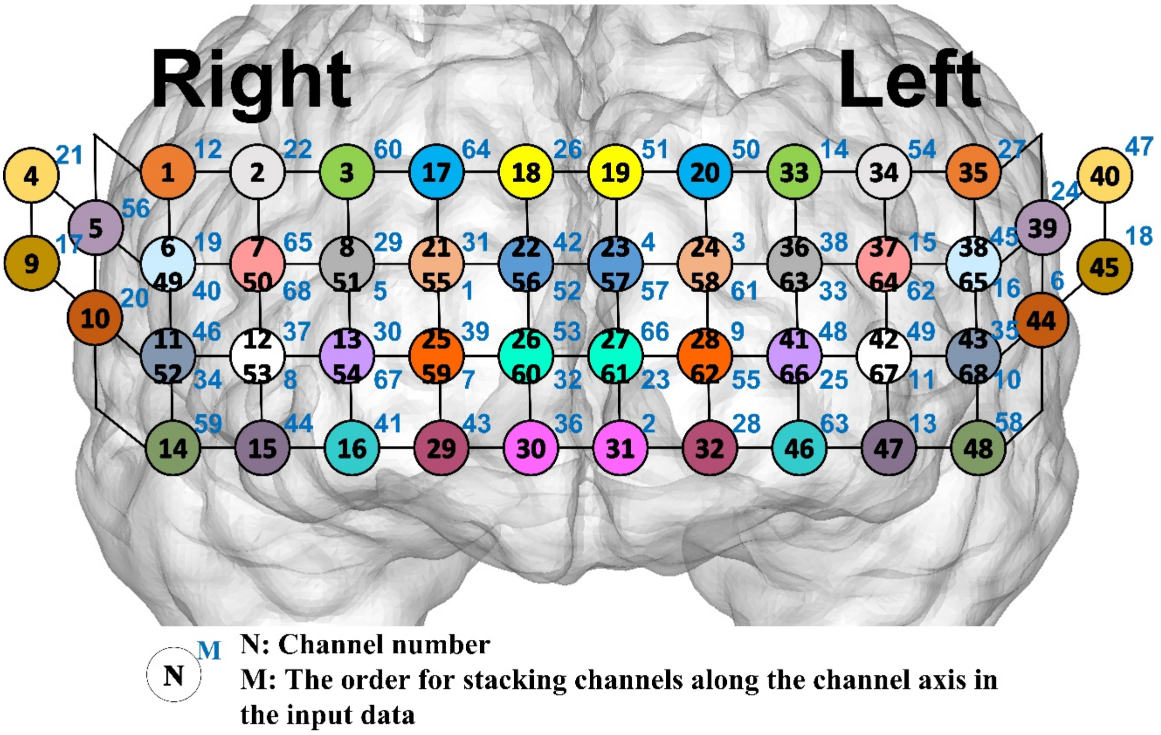

Figure S3. The order for stacking channels along the channel axis in the input data in the C3 paradigm.

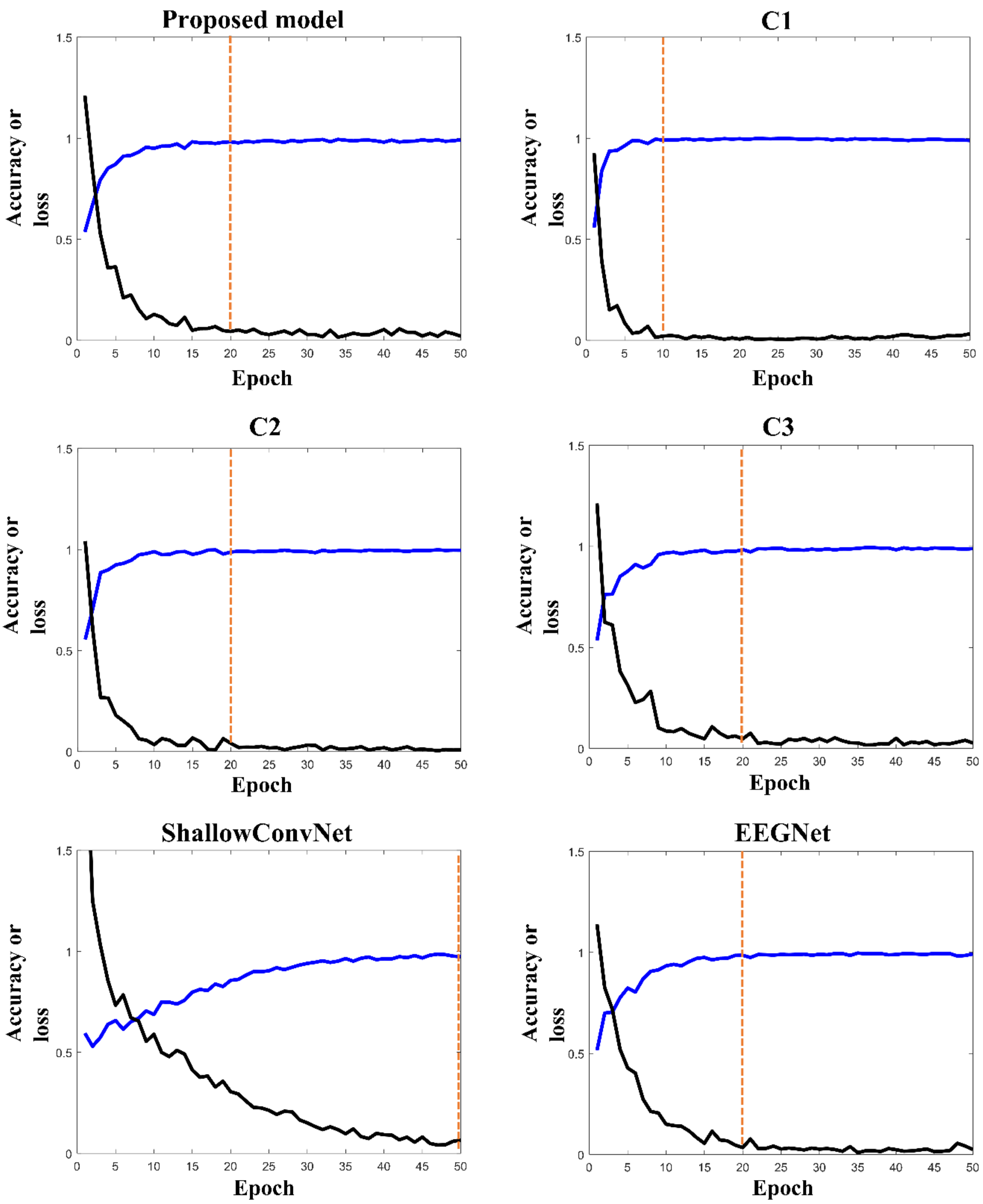

Figure S4. The accuracy according to the number of selected features. The maximum accuracy was shown for the number of features less than 30. Blue line is the training accuracy, black line is the training loss, and yellow dot line is the epoch for test.
